# Myeloma-derived exosomal circ_0002724 promotes osteoclastogenesis through a miR-4753-3p/IFIT1 and RANK axis in myeloma bone disease

**DOI:** 10.3389/fonc.2026.1887386

**Published:** 2026-08-18

**Authors:** Nayan Yi, Muyun Wu, Xiaojie Ma, Yuhan Xie, Heng Sun, Yuxi Zhang, Xing Cui

**Affiliations:** 1 The First School of Clinical Medicine, Shandong University of Traditional Chinese Medicine, Jinan, China; 2 The Second School of Clinical Medicine, Shandong University of Traditional Chinese Medicine, Jinan, China; 3 Center of Oncology and Hematology, The Second Affiliated Hospital of Shandong University of Traditional Chinese Medicine, Jinan, China

**Keywords:** circRNA, multiple myeloma, myeloma bone disease, osteoclast, rank

## Abstract

**Background:**

Myeloma bone disease (MBD) is driven by excessive osteoclast activation and progressive bone destruction, but the role of exosomal circular RNAs (circRNAs) from myeloma cells remains unclear.

**Methods:**

Exosomes from U266 and MM1S cells were isolated and used to treat THP-1 cells. Osteoclast differentiation was assessed by TRAP staining and bone resorption assay. circRNA sequencing of U266 exosomes and MBD patient blood was performed to identify overlapping circRNAs. A ceRNA network was constructed using circBank and miRTarBase, combined with single-cell RNA-seq (GSE271107) to prioritize candidate target genes. Functional experiments included circ_0002724 knockdown/overexpression in U266 cells, exosomal transfer, TRAP, bone resorption, qRT-PCR, RNA-FISH/IF, dual-luciferase reporter assay, and immunohistochemistry of patient bone marrow biopsies.

**Results:**

U266-derived exosomes exhibited stronger osteoclast-inducing activity than MM1S-derived exosomes. Intersection analysis identified 14 common circRNAs, among which circ_0002724 showed the highest predicted miRNA-binding capacity and was significantly upregulated in THP-1 cells after exosome treatment. A circ_0002724-associated regulatory network identified miR-4753-3p, IFIT1, and RANK as potential downstream components. Single-cell analysis revealed that IFIT1 was highly expressed in MM osteoclast precursor cells and increased during osteoclast differentiation. Exosomal circ_0002724 was internalized by THP-1 cells; circ_0002724 knockdown reduced, whereas overexpression enhanced, TRAP-positive osteoclast formation and bone resorption activity, accompanied by altered RANK and IFIT1 expression. Dual-luciferase reporter assays supported the interactions between circ_0002724, miR-4753-3p, RANK, and IFIT1. Immunohistochemical analysis of patient biopsies demonstrated positive associations of circ_0002724, RANK, and IFIT1 with MBD severity.

**Conclusions:**

Myeloma-derived exosomal circ_0002724 promotes osteoclast activation and bone resorption and is associated with regulation of the miR-4753-3p/IFIT1/RANK network. These findings provide new insights into exosome-mediated tumor–bone microenvironment communication and suggest circ_0002724 as a potential biomarker and candidate therapeutic target for MBD.

## 1 Introduction

Multiple myeloma (MM) is the second most common hematologic malignancy and is characterized by clonal proliferation of malignant plasma cells within the bone marrow (1, 2). Despite substantial advances in therapeutic strategies, including proteasome inhibitors, immunomodulatory drugs, monoclonal antibodies, and chimeric antigen receptor T-cell (CAR-T) therapies, MM remains an incurable hematological malignancy associated with considerable mortality. Global cancer statistics estimated approximately 188,000 new MM cases and 121,000 deaths worldwide in 2022, highlighting the persistent disease burden of MM (3).

Multiple myeloma bone disease (MBD) represents one of the most common and debilitating complications of MM, affecting approximately 70–80% of patients during the disease course (4). MBD is characterized by progressive osteolytic destruction, severe bone pain, pathological fractures, and impaired physical function, which substantially compromise patients’ quality of life (5, 6). Although anti-myeloma therapies and bone-targeted treatments have improved clinical outcomes, osteolytic lesions often remain irreversible even in patients achieving hematologic remission (7). Current bone-targeted therapies, including bisphosphonates and denosumab, have significantly improved the management of MBD by reducing skeletal-related events, their clinical application remains limited by potential adverse effects, including medication-related osteonecrosis of the jaw (MRONJ). Moreover, these therapies primarily prevent further skeletal complications rather than fully restore the disrupted bone remodeling process. Therefore, elucidating the molecular mechanisms driving persistent osteoclast activation and identifying novel therapeutic targets remain critical for improving MBD management.

At the cellular level, MBD progression is driven by profound disruption of bone remodeling. Under physiological conditions, bone homeostasis is maintained through the balance between osteoblast (OB)-mediated bone formation and osteoclast (OC)-mediated bone resorption (8). In MM, this balance is shifted toward excessive OC activation accompanied by impaired OB function (9). The receptor activator of nuclear factor-κB (RANK)/receptor activator of nuclear factor-κB ligand (RANKL) pathway represents the central signaling axis regulating osteoclastogenesis. RANKL expressed by OBs, stromal cells, and immune cells binds to RANK on OC precursors and activates downstream signaling cascades, including NF-κB and NFATc1 pathways, thereby promoting OC differentiation, maturation, and bone-resorbing activity (10, 11). However, persistent OC activation in MM cannot be fully explained by increased RANKL signaling alone. Emerging evidence indicates that, in addition to alterations in RANKL availability, intracellular regulatory programs within the MM bone marrow microenvironment may further modulate osteoclast responsiveness and contribute to pathological bone destruction (12, 13).

Beyond soluble cytokine signaling, extracellular vesicle-mediated communication has emerged as an important mechanism by which myeloma cells remodel the bone marrow microenvironment. Myeloma-derived exosomes transfer diverse bioactive cargos to recipient cells within the bone marrow niche and thereby modulate OC differentiation and microenvironment remodeling (14, 15).Circular RNAs (circRNAs) as exosomal cargos, have attracted increasing attention because of their remarkable stability and regulatory potential. Emerging evidence suggests that exosomal circRNAs participate in tumor progression and intercellular communication through competing endogenous RNA (ceRNA) mechanisms (16, 17). Nevertheless, whether myeloma-derived exosomal circRNAs contribute to OC activation through regulation of osteoclastogenic signaling pathways remains poorly understood.

In addition to classical osteoclastogenic signaling, inflammatory regulatory programs have also been implicated in pathological OC activation. Recent studies have suggested that inflammatory mediators and inflammatory microenvironmental remodeling are closely involved in OC differentiation and pathological bone destruction. Dedrobine was reported to suppress OC differentiation and inflammatory bone destruction through regulation of the ROS/NFATc1/MMP9 signaling pathway (18). Complement factor H-related protein 5 was shown to alleviate inflammatory OC differentiation by disrupting RANK-JNK signaling (19). In addition, higenamine was found to regulate macrophage polarization and OC differentiation through THBS-1/TGF-β signaling during inflammatory bone destruction (20). These findings suggest that inflammatory signaling networks may cooperate with osteoclastogenic pathways to sustain pathological bone resorption. However, how tumor-derived exosomal non-coding RNAs connect inflammatory regulatory programs with OC activation in the MM bone marrow microenvironment remains unclear.

Therefore, the present study aimed to investigate whether myeloma-derived exosomal circRNAs contribute to OC activation and MM-associated bone disease, and to explore the potential ceRNA-mediated mechanisms linking exosomal circRNAs to osteoclastogenic and inflammation-related signaling pathways.

## 2 Materials and methods

### 2.1 Cell culture and OC differentiation of THP-1 cells

Human MM cell lines U266 and MM1S, and human monocytic THP-1 cells were obtained from ATCC. U266 and MM1S cells were cultured in RPMI-1640 with 10% FBS and 1% penicillin-streptomycin. THP-1 cells were maintained in RPMI-1640 (Gibco, USA) supplemented with 10% FBS. For OC differentiation, THP-1 cells were seeded at 5×10^4^ cells/well in 24-well plates with 100 ng/mL PMA for 48 h, followed by a 24-h rest in PMA-free medium. Differentiation was induced in α-MEM containing 10% FBS, 25 ng/mL human M-CSF, and 50 ng/mL human sRANKL for 7–14 days, according to a previously described protocol (21).

### 2.2 Exosome isolation and characterization

U266 and MM1S cells were cultured in medium containing exosome-depleted FBS for 48 h. Conditioned media were collected and centrifuged at 800×g for 5 min, 2,000×g 20 min, and 20,000×g for 70 min to remove cells, debris, and larger extracellular vesicles. The supernatants were filtered through 0.22-μm filters and ultracentrifuged at 100,000 × g for 1.5 h. Exosome pellets were resuspended in PBS following a previously reported method (22). The morphology of isolated exosomes was examined by transmission electron microscopy (TEM; JEOL, Japan), and particle size distribution was analyzed by nanoparticle tracking analysis (NTA; Particle Metrix, Germany). The protein concentration of exosomes was quantified using a bicinchoninic acid (BCA) assay.

### 2.3 circRNA sequencing and intersection analysis

Total RNA from U266 exosomes and peripheral blood of MBD patients was extracted, ribosomal RNA removed, and libraries prepared using TruSeq Stranded Total RNA Library Prep Kit (Illumina). Sequencing was performed on Illumina Hiseq. Raw data were processed to identify circRNAs. CircRNAs present in both datasets were selected as candidates.

### 2.4 ceRNA network construction

Candidate circRNAs were subjected to ceRNA network construction to identify potential circRNA–miRNA–mRNA regulatory relationships. Candidate circRNAs were submitted to circBank for prediction of potential miRNA interactions (23). Experimentally validated miRNA–target interactions were obtained from miRTarBase (24), and R software was used to identify downstream target mRNAs of the predicted miRNAs. For circ_0002724, the top 10 miRNAs with the highest regulatory potential were selected based on the number of experimentally validated target genes. The predicted target genes were intersected with curated osteoclast-related gene sets, including interferon-stimulated genes (ISGs), inflammatory genes, osteoclast transcription factors, macrophage polarization markers (M1/M2), and TNFRSF11A (RANK)-related genes. Candidate regulatory axes identified from the ceRNA network were subsequently evaluated using single-cell RNA-seq analysis and experimental assays.

### 2.5 Single-cell RNA-seq analysis

GSE271107 dataset was downloaded from GEO. Data processing, cell type annotation, differential expression (Wilcoxon test, *|log2FC|*>0.25, *p.adj* < 0.05), pseudotime analysis (Monocle), and UMAP visualization were performed using Seurat (v4) and Monocle (v2) in R. The major cell populations in the dataset were annotated according to the original annotation information provided by GSE271107, including malignant myeloma cells, B cells, T cells, NK cells, myeloid cells, neutrophils, dendritic cells, erythroid cells, hematopoietic progenitor cells, stromal cells, OBs, and OC precursors. The Osteoclast_precursor cluster was further defined based on the expression of canonical osteoclast lineage-associated markers, including CSF1R and TNFRSF11A (RANK).

### 2.6 Functional assays in THP-1 cells

THP-1 macrophages were treated with: (1) negative control (no M-CSF/sRANKL), (2) positive control (M-CSF/sRANKL), (3) U266 conditioned medium (10% v/v), (4) GW4869-pretreated U266 CM, (5) U266 exosomes (20 μg/mL), (6) exosomes from circ_0002724-knockdown (siRNA) or overexpressing (plasmid) U266 cells, and (7) corresponding controls. For GW4869 pretreatment, U266 cells were incubated with GW4869 (5 μM; Selleck, USA) or an equivalent volume of DMSO for 24 h to inhibit exosome release, as previously described by Zhang (25). The conditioned medium was then collected for subsequent experiments.

### 2.7 TRAP staining

After 7–14 days of induction, cells were washed with PBS and fixed with 4% paraformaldehyde (Sigma, USA) for 10 min at room temperature. After fixation, cells were permeabilized with cold 50% acetone/50% methanol for 5 min. TRAP staining was performed using a commercial TRAP assay kit (Sigma, USA) according to the manufacturer’s instructions, as previously described (26). OCs were identified as TRAP-positive multinucleated cells containing three or more nuclei. Stained cells were observed and photographed under a light microscope (Nikon, Japan). For quantification, at least five randomly selected fields per well were counted, and the number of TRAP-positive multinucleated cells per well was recorded. Hematoxylin was used to counterstain nuclei when necessary. The relative number of OCs was normalized to the positive control group (M-CSF + sRANKL only), all experiments were independently performed three times.

### 2.8 Bone resorption assay

THP-1 cells were seeded onto sterilized bovine bone slices and treated under different experimental conditions to induce OC differentiation. After 14 days of induction, osteoclasts attached to the bone slices were removed using 0.25% EDTA-trypsin. The bone slices were subsequently dehydrated and subjected to gold sputter coating. Osteoclast-mediated bone resorption pits were visualized using scanning electron microscopy (SEM; HITACHI S4800, Tokyo, Japan). Representative SEM images were acquired, and the percentage of resorbed area was quantified using ImageJ software (NIH, Bethesda, MD, USA) by calculating the resorbed area relative to the total analyzed bone surface area. All experiments were independently performed three times.

### 2.9 Quantitative real-time PCR (qRT-PCR)

Total RNA was extracted using TRIzol reagent (Invitrogen, USA) according to the manufacturer’s instructions. cDNA was synthesized using a reverse transcription kit, and quantitative real-time PCR was performed using SYBR Green Master Mix (Applied Biosystems, USA). Relative gene expression levels were normalized to β-actin and calculated using the 2^-ΔΔCt method. The primer sequences were as follows: circ_0002724 forward: 5′-AGTGGTGCAACTTCCTCCTTC-3′, reverse: 5′-CCAAGTGCAGCTCCCATTCT-3′; β-actin forward: 5′-CCCTATAAAACCCAGCGGCG-3′, reverse: 5′-TCGTCGCCCACATAGGAATC-3′; RANK forward: 5′-CAGCTAATTTGTGGCACTGG-3′, reverse: 5′-ACCTGAGGACTCCTTATCTCCA-3′; IFIT1 forward: 5′-GGCTATGGCTCATCCAGTCA-3′, reverse: 5′-ATCTCCTGCCAGCAAGAAGA-3′. All primers were synthesized by Dingguo Biotechnology (Beijing, China).

### 2.10 Fluorescence *in situ* hybridization combined with immunofluorescence (FISH-IF)

RNA fluorescence *in situ* hybridization (RNA-FISH) was performed using probes targeting the back-splice junction of circ_0002724, generated by *in vitro* transcription using primers containing a T7 promoter (forward: TAATACGACTCACTATAGGGAGGGTTCGGGAAGCTGTTGGAGC; reverse: CCATCGTCATCATCCTCTGAC). Hybridization was carried out at 37 °C overnight, followed by washing with SSC buffers. For U266 cells, cells were fixed with 4% paraformaldehyde, permeabilized with 0.3% Triton X-100, and blocked with 5% bovine serum albumin (BSA). After RNA-FISH, cells were incubated with an anti-CD63 antibody followed by a fluorescent secondary antibody. Nuclei were counterstained with DAPI, and images were acquired using a confocal microscope (Leica TCS SP8). For bone marrow tissue sections, formalin-fixed paraffin-embedded (FFPE) sections (4 μm) were deparaffinized, rehydrated, and subjected to antigen retrieval in citrate buffer (pH 6.0, 95 °C, 15 min). After blocking, sections were hybridized with the circ_0002724 probe and subsequently incubated with anti-CD138 antibody (1:200, Abcam) followed by Cy3-conjugated secondary antibody (1:500). Nuclei were stained with DAPI, and images were captured using a confocal microscope.

### 2.11 Dual-luciferase reporter assay

Wild-type or mutant circ_0002724 sequences and IFIT1 3′UTR fragments containing the predicted miR-4753-3p binding sites were cloned into luciferase reporter vectors. HEK293T cells were co-transfected with reporter constructs and miR-4753-3p mimic or negative control using Lipofectamine 3000 (Invitrogen, USA). After 48 h, firefly and renilla luciferase activities were measured using a dual-luciferase reporter assay system according to the manufacturer’s instructions.

### 2.12 IHC staining

Immunohistochemical staining was performed on paraffin-embedded human bone marrow biopsy sections. Sections were deparaffinized, rehydrated through graded ethanol solutions, and subjected to heat-induced antigen retrieval. Endogenous peroxidase activity was blocked with hydrogen peroxide, followed by blocking of nonspecific binding. Sections were incubated with primary antibodies overnight at 4 °C, followed by incubation with HRP-conjugated secondary antibodies. Immunoreactive signals were visualized using a DAB chromogenic substrate, and nuclei were counterstained with hematoxylin. Images were captured using a light microscope, and immunoreactive scores were calculated based on staining intensity and the proportion of positive cells. Primary antibodies included anti-RANK antibody (Abcam, UK) and anti-IFIT1 antibody (Abcam, UK).

### 2.13 Clinical study design and patient assessment

A retrospective study was conducted to analyze newly diagnosed multiple myeloma (MM) patients with myeloma bone disease (MBD), aiming to investigate the association between MBD severity and the expression levels of circ_0002724 and RANK. This study was approved by the Ethics Committee of the Second Affiliated Hospital of Shandong University of Traditional Chinese Medicine (2023 Ethical Review-KY-024-01), and written informed consent was obtained from all patients.

All patients were recruited and treated at the same institution. Bone marrow biopsies from newly diagnosed MM patients with MBD were collected. IHC was performed using anti-RANK and IFIT1 antibody; FISH-IF for circ_0002724 and CD138 was done as described. MBD severity was assessed by radiological scoring.

### 2.14 Statistical analysis

The experimental results were subjected to statistical analysis using SPSS 25.0 and GraphPad Prism 10.0. Data are presented as mean ± SD. Comparisons between two groups used t-test; multiple groups used one-way ANOVA. Spearman’s rank correlation analysis was used to assess the associations among MBD severity, circ_0002724 FISH expression, RANK IHC score, and IFIT1 IHC score. All tests were two-sided, with *P* < 0.05 considered statistically significant.

## 3 Results

### 3.1 U266-derived exosomes exhibit superior OC-inducing capacity

**Figure 1:**
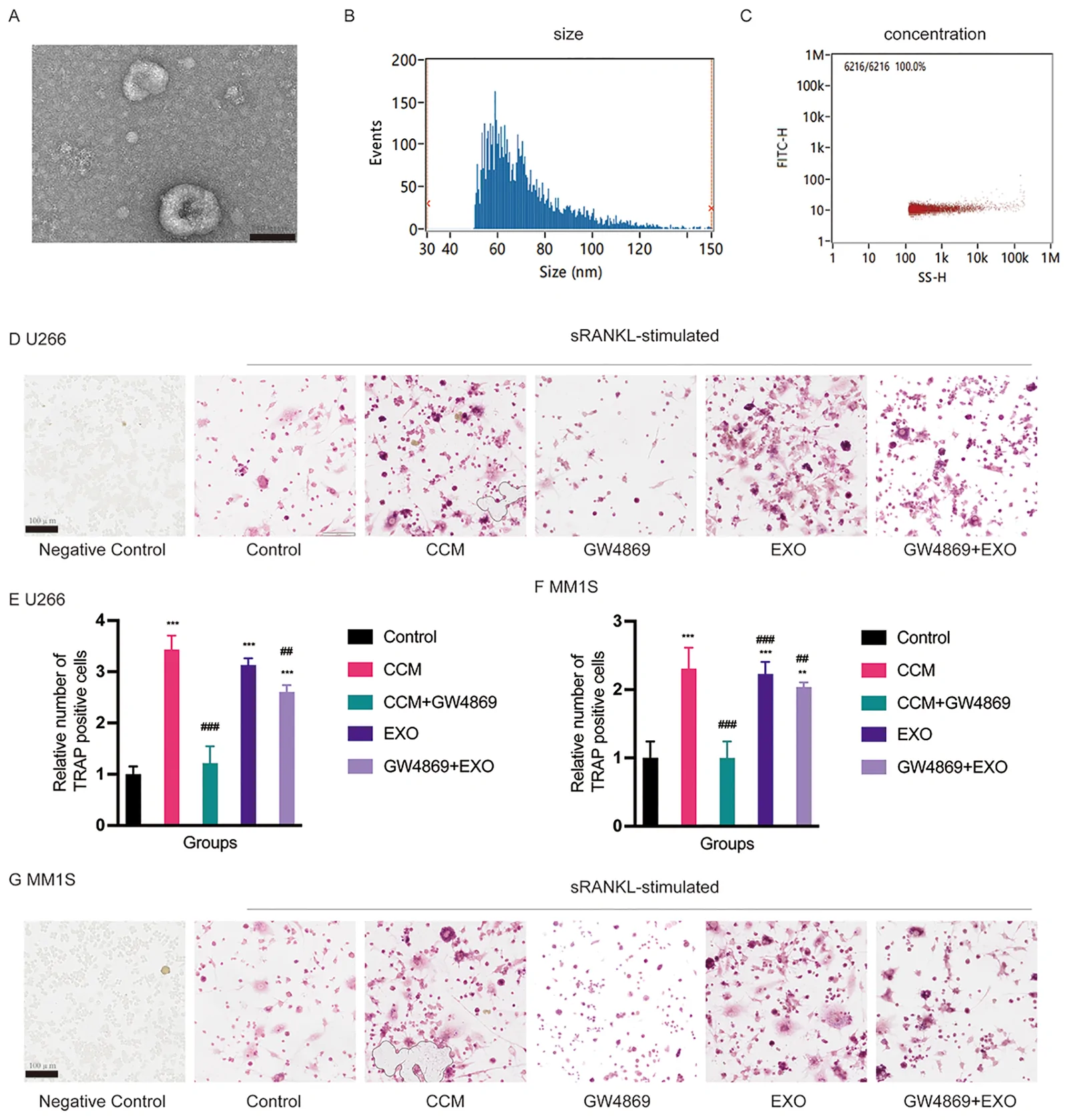
U266-derived exosomes exhibit superior osteoclast-inducing capacity. Morphology and size distribution of U266-derived exosomes were verified by transmission electron microscopy (TEM). Scale bar = 100nm. **(B)** Nanoparticle tracking analysis (NTA) showing the size distribution of U266-derived exosomes. **(C)** Flow cytometry analysis of U266-derived exosomes. **(D, G)** Representative images of TRAP staining showing osteoclast differentiation in THP-1 cells stimulated with sRANKL under different treatments: Negative Control (no treatment), Control (sRANKL alone), CCM (MM-conditioned medium), GW4869 (exosome secretion inhibitor-treated CCM), EXO (purified U266-derived exosomes), and GW4869+EXO (GW4869 plus purified exosomes). Scale bar = 100 μm. **(E, F)** Quantification of TRAP-positive multinucleated osteoclasts in THP-1cells with U266-derived **(E)** and MM1S-derived **(F)** exosomes. Data are presented as the mean ± SD. ***p < 0.001 vs. Control group; ###p < 0.001 vs. GW4869 group.

To investigate the role of myeloma-derived exosomes in OC activation, exosomes were isolated from U266 and MM1S cells and characterized by transmission electron microscopy (TEM) and nanoparticle tracking analysis (NTA) (**Figures 1A–C**). Both exosome preparations exhibited typical cup-shaped morphology with a mean diameter of approximately 100 nm. When added to THP-1-derived OC precursors, exosomes from both cell lines promoted the formation of TRAP-positive multinucleated cells. Notably, U266 exosomes induced a significantly higher number of OCs compared with MM1S exosomes (**Figures 1D–G**), indicating that U266 cells possess a stronger exosome-mediated osteoclastogenic capacity.

### 3.2 Identification of exosomal circ_0002724 as a candidate promoter of osteoclastogenesis in multiple myeloma

To identify functionally relevant exosomal circRNAs, we performed circRNA sequencing on U266-derived exosomes and on peripheral blood from MBD patients.

**Figure 2:**
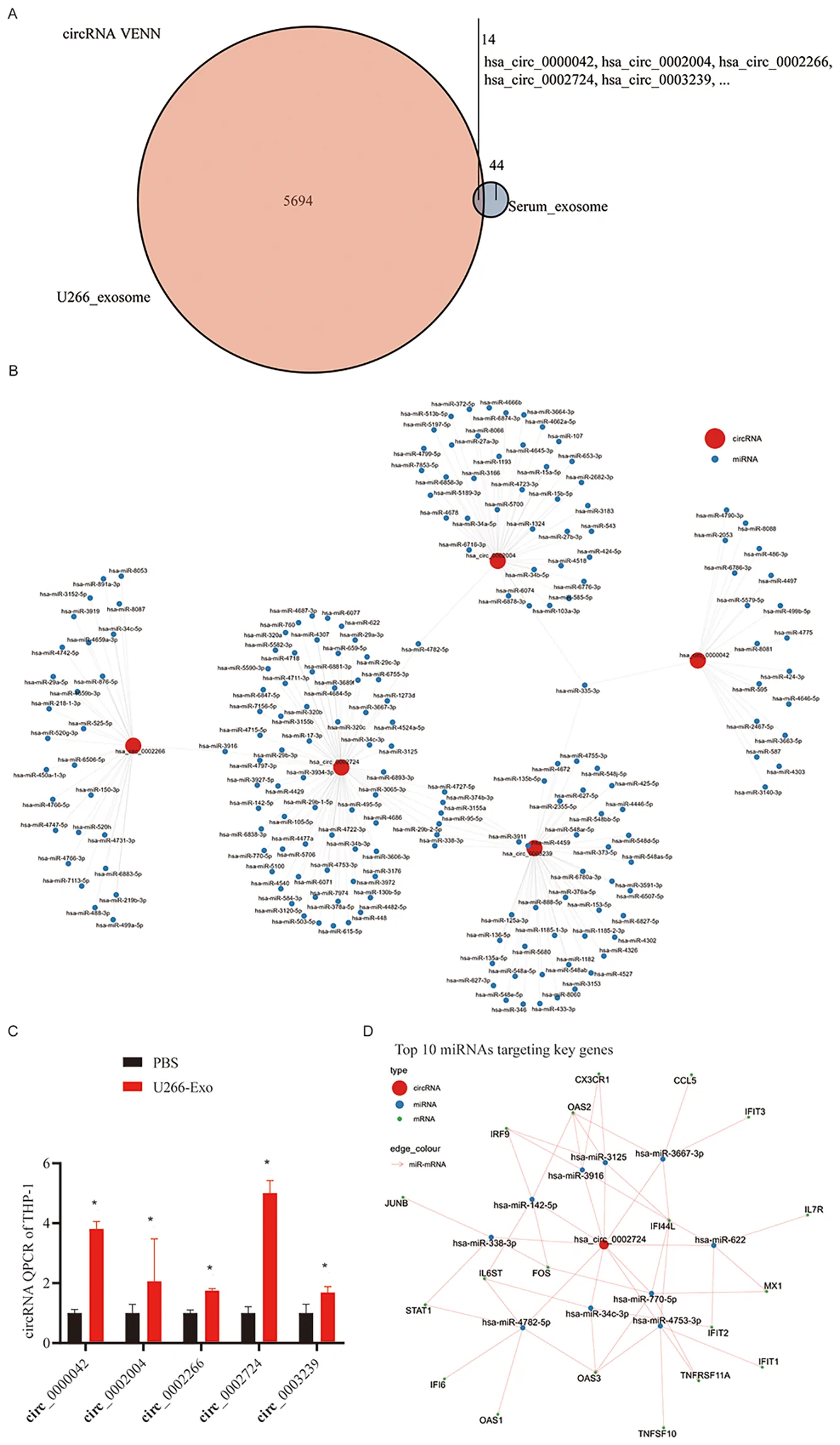
Identification of exosomal circ_0002724 as a candidate promoter of osteoclastogenesis in multiple myeloma. **(A)** Venn diagram showing the overlap of 14 main circRNAs detected in U266 cell-derived exosomes and serum exosomes and listed circ_0002724. **(B)** ceRNA network analysis of the top five exosomal circRNAs, illustrating their potential interactions with target miRNAs and downstream mRNAs. Red nodes represent circRNAs, and blue nodes represent mRNAs. **(C)** RT-qPCR validation of the expression levels of five candidate circRNAs in THP-1 cells treated with PBS or U266-derived exosomes. *p < 0.05 vs. PBS group. **(D)** Regulatory network showing the top 10 miRNAs targeting key osteoclast-related genes, centered on circ_0002724. Red nodes represent circRNA, blue nodes represent miRNAs, and green nodes represent mRNAs. The edge color indicates the interaction relationship.

Intersection analysis revealed 14 circRNAs present in both datasets (**Figure 2A**). Using circBank and miRTarBase, we predicted miRNA-binding capacities for these circRNAs. Among the top five circRNAs with the highest number of potential miRNA interactions, circ_0002724 (hsa_circ_0002724) exhibited the largest miRNA-sponging potential (**Figure 2B**). We then treated THP-1 cells with U266 exosomes and measured the expression levels of these five circRNAs by qRT-PCR before and after treatment. Compared with untreated cells, circ_0002724 showed the most significant increase after exosome exposure (**Figure 2C**). Therefore, we focused on circ_0002724 for further analysis. Many circRNAs have been reported to regulate gene expression through competing endogenous RNA (ceRNA) mechanisms by acting as miRNA sponges, thereby influencing downstream target genes involved in tumor progression and microenvironment remodeling (27). Therefore, we constructed a ceRNA network to explore potential regulatory mechanisms underlying the role of exosomal circ_0002724 in MM-associated bone disease.

We selected the top 10 miRNAs predicted to bind circ_0002724 based on the number of experimentally validated target genes. To identify MBD-relevant downstream candidates, these predicted target genes were further integrated with osteoclast-related gene sets, including genes involved in osteoclastogenesis, inflammatory regulation, interferon responses, macrophage polarization, and RANK-associated signaling. These biological processes were selected because they represent key regulatory programs involved in pathological osteoclast activation and bone destruction in MM.

The resulting network highlighted the circ_0002724/miR-4753-3p/IFIT1 and circ_0002724/miR-4753-3p/TNFRSF11A (RANK) axes (**Figure 2D**), which were subsequently investigated by single-cell RNA-seq analysis and experimental validation.

### 3.3 Single-cell transcriptomics identifies IFIT1 as a top upregulated gene in MM osteoclast precursors and links it to aberrant differentiation

**Figure 3:**
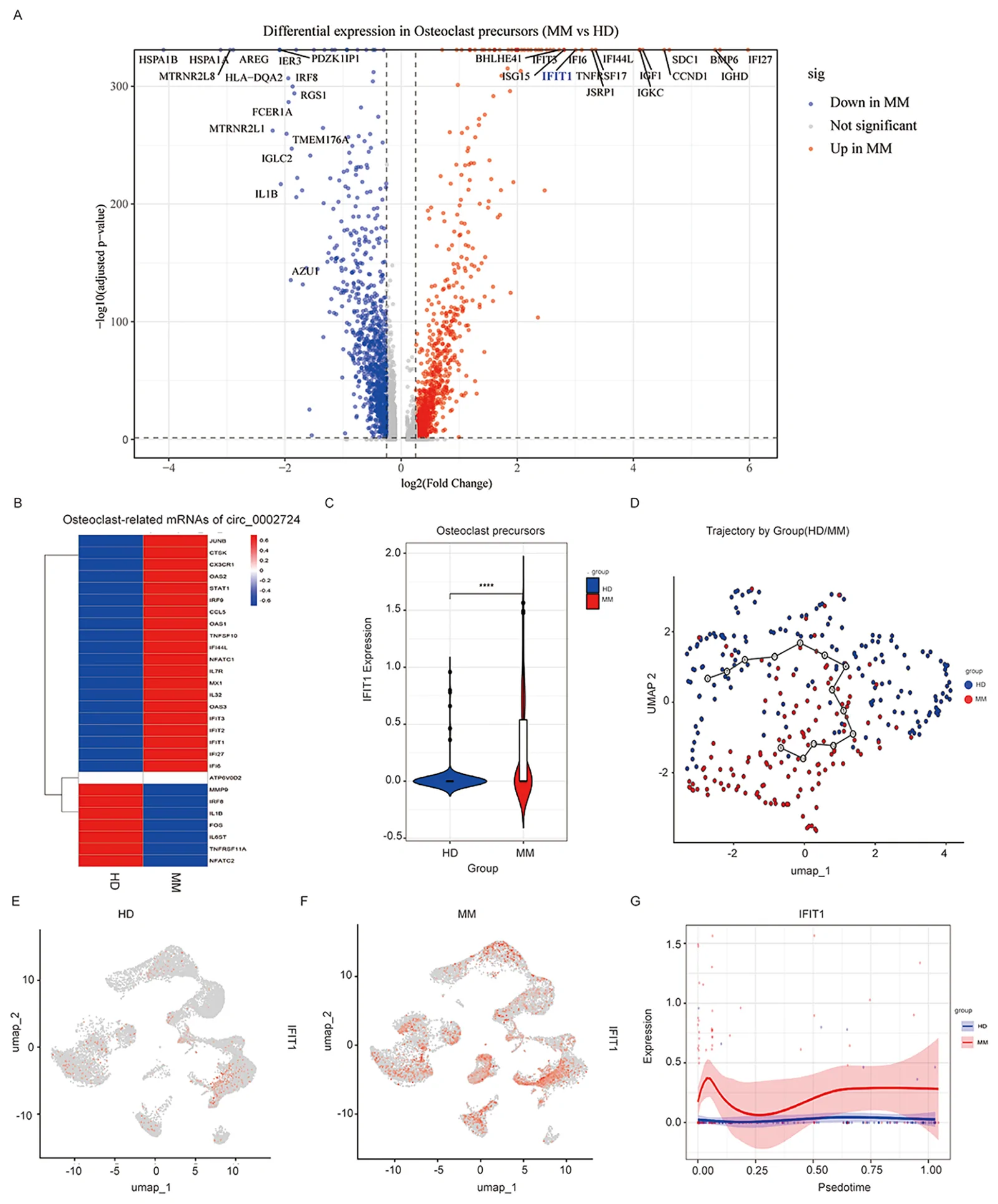
Single-cell transcriptomics identifies IFIT1 as a top upregulated gene in MM osteoclast precursors and links it to aberrant differentiation. **(A)** Volcano plot showing differentially expressed genes (DEGs) in OC precursors from MM patients versus healthy donors (HD). Significantly upregulated and downregulated genes are highlighted in red and blue, respectively; IFIT1 is labeled as a top upregulated gene. **(B)** Heatmap displaying the expression pattern of osteoclast-related mRNAs potentially targeted by circ_0002724 in HD and MM groups. Red indicates high expression, blue indicates low expression. **(C)** Violin plot comparing IFIT1 expression levels in OC precursors between HD and MM samples. ****p < 0.0001. **(D)** Pseudotime trajectory analysis of OC precursors colored by group (HD/MM). **(E, F)** UMAP visualization of IFIT1 expression distribution in OC precursors from HD **(E)** and MM **(F)** samples. **(G)** Pseudotime expression dynamics of IFIT1 along the osteoclast differentiation trajectory in HD and MM groups.

To explore the clinical relevance of the predicted targets, we analyzed the single-cell RNA-seq dataset GSE271107, which includes bone marrow samples from MM patients and healthy donors (HD). Focusing on OC precursors (annotated as “osteoclast _precursor”), we performed differential expression analysis between MM and HD. Among the top 15 upregulated genes in MM precursors, IFIT1 was consistently ranked high (**Figures 3A, B**). Notably, IFIT1 was also a predicted target of circ_0002724 in our ceRNA network. Violin plots confirmed that IFIT1 expression was significantly higher in MM-derived OC precursors compared with HD (**Figure 3C**). UMAP feature plots showed broader and stronger IFIT1 expression in MM samples, particularly within the OC precursors cluster (**Figures 3D, E**). Pseudotime trajectory analysis was performed to reconstruct the differentiation path of OC precursors. The results revealed that MM cells exhibited a more dispersed and aberrant trajectory compared with HD cells (**Figure 3F**). Along the pseudotime axis, IFIT1 expression increased progressively in both groups, but remained consistently higher in MM cells throughout differentiation (**Figure 3G**). This sustained elevation of IFIT1 in MM precursors suggests that it may contribute to the enhanced OC activation seen in MBD. Given that IFIT1 is a key interferon-stimulated gene, its aberrant upregulation likely reflects an inflammatory state in MM precursors, driving uncontrolled OC differentiation and bone destruction.

### 3.4 Exosomal circ_0002724 is internalized by THP-1 cells and promotes OC activation and bone resorption through the miR-4753-3p/RANK/IFIT1 regulatory axis

**Figure 4:**
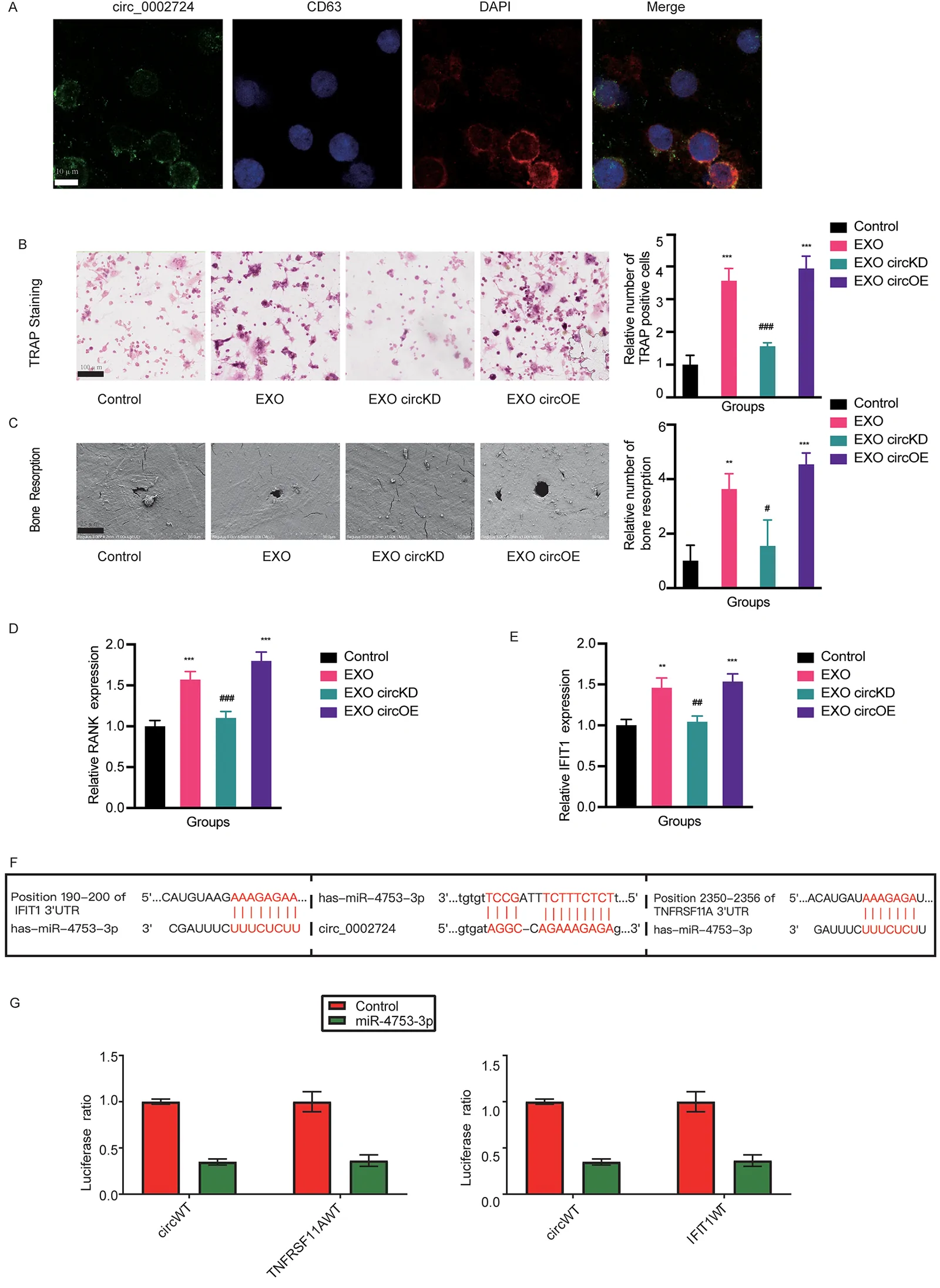
Exosomal circ_0002724 is internalized by THP-1 cells and promotes OC activation and bone resorption through the miR-4753-3p/RANK/IFIT1 regulatory axis. **(A)** Representative images of RNA fluorescence *in situ* hybridization combined with immunofluorescence (FISH-IF) showing the internalization of exosomal circ_0002724 in THP-1 cells. circ_0002724 (green), exosomal marker CD63 (red), and nuclei (DAPI, blue). Scale bar = 10 μm. **(B)** TRAP staining of THP-1-derived osteoclasts treated with control exosomes, exosomes from circ_0002724-knockdown (EXO circKD) or overexpressing (EXO circOE) U266 cells, and corresponding quantitative analysis of TRAP-positive multinucleated cells. Scale bar = 100 μm. ***p < 0.001 vs. Control; ###p < 0.001 vs. EXO group. **(C)** Bone resorption assay of osteoclasts under different treatments, with corresponding quantification of bone resorption pits. *p < 0.05, **p < 0.01 vs. Control; #p < 0.05 vs. EXO group. **(D, E)** RT-qPCR analysis showing the relative mRNA expression of RANK **(D)** and IFIT1 **(E)** in THP-1 cells treated with different exosomes. **p < 0.01, ***p < 0.001 vs. Control; ##p < 0.01, ###p < 0.001 vs. EXO group. **(F)** Schematic representation of the predicted binding sites among circ_0002724, hsa-miR-4753-3p, and the 3’UTRs of IFIT1 and TNFRSF11A (RANK). **(G)** Dual-luciferase reporter assay verifying the direct interactions between hsa-miR-4753-3p and circ_0002724, as well as between hsa-miR-4753-3p and the 3’UTRs of IFIT1 and TNFRSF11A.

To confirm that circ_0002724 is indeed transferred via exosomes, we performed RNA-FISH for circ_0002724 combined with immunofluorescence for the exosomal marker CD63 in U266 cells. Circ_0002724 signals co-localized with CD63, indicating its packaging into exosomes. When THP-1 cells were incubated with U266-derived exosomes, circ_0002724 signals appeared in the cytoplasm of recipient cells, confirming internalization (**Figure 4A**).

We then generated U266 cells with circ_0002724 knockdown (circKD) or overexpression (circOE) using specific siRNA or plasmid. Exosomes isolated from these cells were used to treat THP-1 cells in four groups: control (PBS), exosome (EXO), circKD-exosome (circKD), and circOE-exosome (circOE). TRAP staining showed that EXO significantly increased the number of TRAP-positive multinucleated cells compared with control. This effect was attenuated by circKD and enhanced by circOE (**Figure 4B**). Bone resorption assay on bovine bone slices yielded consistent results: resorption pit area was reduced by circKD and increased by circOE relative to EXO (**Figure 4C**). qRT-PCR analysis revealed that EXO upregulated the mRNA levels of RANK and IFIT1 in THP-1 cells, and this effect was further augmented by circOE and diminished by circKD (**Figures 4D, E**). To investigate the underlying regulatory mechanism, bioinformatic analyses based on circBase and TargetScan predicted complementary binding sites between miR-4753-3p and circ_0002724 as well as the 3′UTR of IFIT1, supporting a potential circ_0002724/miR-4753-3p/IFIT1 axis (**Figure 4F**). Based on the predicted ceRNA interactions, we performed dual-luciferase reporter assays to validate direct binding. Co-transfection of miR-4753-3p mimic significantly suppressed the luciferase activity of reporter constructs containing wild-type circ_0002724 or the 3′UTR of RANK or IFIT1, whereas mutation of the predicted binding sites abolished this suppression (**Figures 4G, H**). These results suggest that circ_0002724 may regulate RANK and IFIT1 through a miR-4753-3p-dependent mechanism.

### 3.5 Clinical correlations of circ_0002724, RANK, and IFIT1 with MBD severity in patient bone marrow biopsies

**Figure 5:**
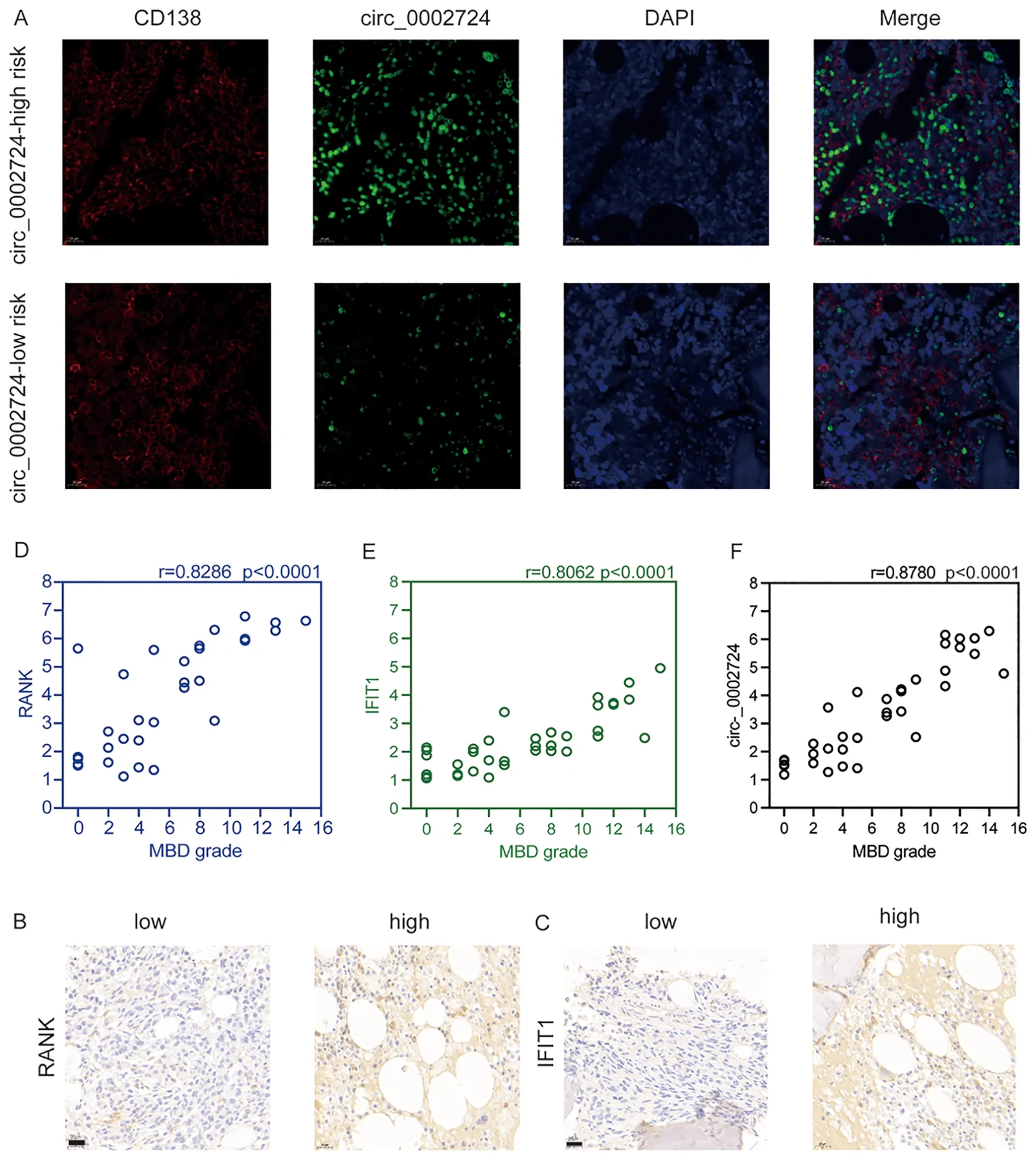
Clinical correlations of circ_0002724, RANK, and IFIT1 with MBD severity in patient bone marrow biopsies. **(A)** Representative images of RNA fluorescence *in situ* hybridization combined with immunofluorescence (FISH-IF) for circ_0002724 (green) and the myeloma cell marker CD138 (red) in bone marrow biopsies from patients with high- and low-risk MBD. Nuclei were stained with DAPI (blue). **(B, C)** Immunohistochemical (IHC) staining of RANK **(B)** and IFIT1 **(C)** in bone marrow biopsies from patients with low and high MBD severity. Scale bars as indicated. **(D)** Spearman correlation between RANK expression and MBD severity in bone marrow biopsy samples from MM patients (r = 0.8286, p<0.0001). **(E)** Spearman correlation between IFIT1 expression and MBD severity in bone marrow biopsy samples from MM patients (r = 0.8062, p<0.0001). **(F)** Spearman correlation between circ_0002724 expression and MBD severity in bone marrow biopsy samples from MM patients (r = 0.8780, p<0.0001).

To evaluate the clinical significance of our findings, we performed immunohistochemical (IHC) staining for RANK and IFIT1, and fluorescence *in situ* hybridization (FISH) for circ_0002724, on bone marrow biopsies from a cohort of newly diagnosed MM patients with MBD (n=36). MBD severity was assessed radiologically and graded as mild or severe. FISH revealed that circ_0002724 expression was significantly higher in patients with severe MBD compared with those with mild MBD (**Figure 5A**). IHC analysis demonstrated that both RANK and IFIT1 protein levels were also elevated in severe MBD patients (**Figures 5B, C**). Spearman correlation analysis demonstrated significant positive associations between MBD severity and the expression levels of RANK, IFIT1, and circ_0002724. RANK and IFIT1 expression levels were positively correlated with MBD grade (RANK: r = 0.8286, p < 0.0001; IFIT1: r = 0.8062, p < 0.0001), while circ_0002724 expression showed a strong positive correlation with MBD grade (r = 0.8780, p < 0.0001) (**Figures 5D–F**). These data indicate that circ_0002724, RANK, and IFIT1 are clinically relevant biomarkers for MBD severity.

### 3.6 Host gene HUWE1 expression in single-cell data

**Figure 6:**
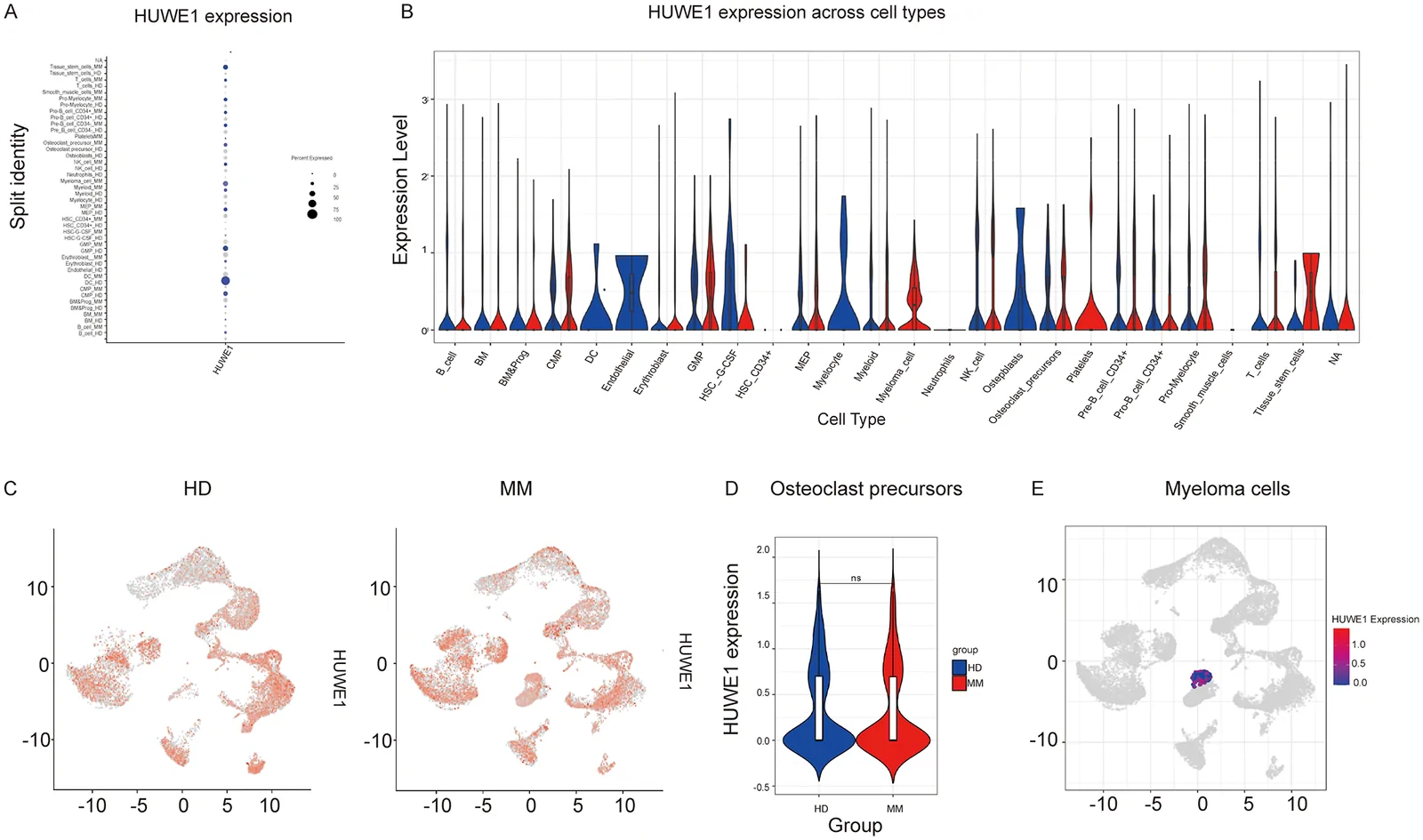
Host gene HUWE1 expression in single-cell data. **(A)** Dot plot showing HUWE1 expression across different cell populations in bone marrow samples from healthy donors (HD) and multiple myeloma (MM) patients. The size of each dot represents the percentage of cells expressing HUWE1, and color intensity indicates the expression level. **(B)** Violin plot illustrating HUWE1 expression levels across major bone marrow cell types in HD (blue) and MM (red) samples. **(C)** UMAP visualization of HUWE1 expression distribution in bone marrow cells from HD and MM samples. Cells are colored by HUWE1 expression level. **(D)** Violin plot comparing HUWE1 expression levels in OC precursors between HD and MM samples (ns, not significant). **(E)** UMAP plot highlighting HUWE1 expression specifically within the myeloma cell population in MM bone marrow samples.

circ_0002724 is derived from the HUWE1 gene locus (exons). To understand the cellular distribution of its host gene, we analyzed HUWE1 expression in the GSE271107 single-cell dataset. HUWE1 was broadly expressed across multiple cell types in both HD and MM bone marrows, including endothelial cells, tissue stem cells, myeloma cells, and OC precursors (**Figures 6A, B**). UMAP visualization showed that HUWE1 was present in OC precursors but did not exhibit a clear differential expression pattern between MM and HD in this cell population (**Figure 6C**). Quantitative comparison confirmed no significant difference in HUWE1 expression in OC precursors between the two groups (**Figure 6D**). However, HUWE1 showed a more clustered expression pattern within myeloma cell clusters, suggesting that the host gene might primarily function within tumor cells rather than directly modulating OC differentiation. Nevertheless, the fact that circ_0002724 is derived from HUWE1 and its exosomal transfer promotes OC activation indicates that the circular RNA can act independently of its linear host transcript.

## 4 Discussion

In the present study, we demonstrated that MM-derived exosomal circ_0002724 promotes OC activation and bone resorption through a ceRNA-mediated regulatory mechanism involving miR-4753-3p, RANK, and IFIT1. Functionally, circ_0002724 overexpression enhanced OC differentiation and increased RANK and IFIT1 expression, whereas circ_0002724 knockdown attenuated these effects. Dual-luciferase reporter assays further supported the direct interaction between circ_0002724, miR-4753-3p, and its downstream targets RANK and IFIT1. However, direct miR-4753-3p rescue experiments were not performed, and further validation is required to confirm the functional contribution of miR-4753-3p within this regulatory network. Single-cell transcriptomic analyses identified IFIT1 as a dynamically upregulated gene during OC differentiation in MM samples, while clinical analyses demonstrated positive associations among circ_0002724, RANK, IFIT1, and MBD severity. In addition, analysis of the host gene HUWE1 suggested that the observed biological effects were more likely associated with circ_0002724 itself rather than alterations in host gene transcription. Collectively, these findings suggest that exosomal circ_0002724 may participate in MBD progression by enhancing OC activation through coordinated regulation of RANK-associated osteoclastogenic signaling and IFIT1-related inflammatory responses.

Accumulating evidence has highlighted the importance of exosome-mediated communication in bone disease. Bone marrow mesenchymal stem cell-derived exosomes have been reported to promote bone regeneration, and Fe3O4 nanoparticles were shown to further enhance this effect through miR-429-mediated inhibition of NOG, alleviation of oxidative stress, and restoration of osteogenic differentiation in irradiated BMSCs (28). In addition, preclinical studies demonstrated that extracellular vesicle administration reduced femoral head necrosis, improved trabecular structure, and enhanced neovascularization (29, 30). However, in the context of MM-associated bone disease, exosome-mediated mechanisms that sustain osteoclastogenic signaling remain incompletely understood. Previous studies investigating non-coding RNAs in osteoclastogenesis mainly focused on miRNAs and lncRNAs, whereas the contribution of exosomal circRNAs to persistent osteoclastogenic signaling in MM has remained largely unclear. Yu et al. demonstrated that MM-derived exosomal ERK1 promotes OC differentiation and participates in MM-associated bone disease progression (22). We further identified circ_0002724 as a functional exosomal circRNA associated with OC activation through regulation of the RANK and IFIT1-related pathways, suggesting a potential ceRNA-associated mechanism in MM bone disease. These findings extend the current understanding of exosomal circRNA-mediated communication within the MM bone microenvironment. Inflammatory signaling represents an important component of pathological bone remodeling. In periodontitis, inflammatory cytokine release promotes OC differentiation and accelerates periodontal bone destruction (31). Similarly, regulation of inflammatory pathways has been shown to influence osteoclast activity and bone loss in osteoporosis-related models (32).

IFIT1 is a classical interferon-stimulated gene involved in innate immune and interferon-related responses. Although anti-inflammatory properties of IFIT1 have been reported in respiratory diseases (33, 34), atherosclerosis (35), and viral infections (36), its role in MM-associated bone disease remains poorly characterized. Lu et al. identified IFIT1 + neutrophil subset amplifying inflammatory responses and inducing hepatocyte apoptosis (37), Xue et al. demonstrated that IFIT1 promotes RANKL-induced OC formation through activation of the JAK1/STAT3 pathway and increases the expression of osteoclast -related markers, whereas IFIT1 silencing attenuated OC differentiation and bone resorption activity (38). Consistent with these findings, our study observed increased IFIT1 expression during OC differentiation, accompanied by enhanced RANK expression. Furthermore, circ_0002724 modulation altered IFIT1 and RANK expression levels, suggesting that IFIT1 may serve as an inflammatory regulatory component linking tumor-derived exosomal signaling with osteoclastogenic responses in MBD.

Several limitations should be acknowledged. First, although our *in vitro* experiments and patient-based analyses support the role of exosomal circ_0002724 in regulating OC activation, an *in vivo* model was not established in this study. Therefore, the systemic distribution, bone-targeting effects, and dynamic regulation of circ_0002724-containing exosomes within the MM microenvironment remain unclear. Whether circ_0002724 inhibition can effectively alleviate bone loss or improve MBD-related outcomes requires further validation using appropriate animal models, such as exosome administration models or MM xenograft models. Second, although dual-luciferase assays supported the regulatory relationship among circ_0002724, miR-4753-3p, RANK, and IFIT1, direct miR-4753-3p rescue experiments were not performed, and future studies are required to further confirm the functional role of miR-4753-3p in this regulatory axis. Finally, although IFIT1 was identified as a potential downstream regulatory molecule associated with OC activation, the precise molecular mechanisms through which IFIT1 integrates inflammatory signaling and osteoclastogenic programs within the MM microenvironment require further investigation.

In conclusion, our study identifies MM-derived exosomal circ_0002724 as a novel regulator of OC activation and bone resorption. Mechanistically, circ_0002724 is incorporated into exosomes and transferred to OC precursors, where it is associated with the regulation of the miR-4753-3p/RANK/IFIT1 axis, leading to enhanced RANK-related osteoclastogenic signaling and IFIT1-associated inflammatory responses. These findings provide new insights into exosome-mediated tumor–bone microenvironment communication in MBD and suggest that circ_0002724 and its downstream regulatory network may serve as potential biomarkers and candidate therapeutic targets for MM-associated bone disease, warranting further validation *in vivo*.

## Data Availability

The raw data supporting the conclusions of this article will be made available by the authors, without undue reservation.
